# Intensity-Modulated Radiation Therapy with Stereotactic Body Radiation Therapy Boost for Unfavorable Prostate Cancer: The Georgetown University Experience

**DOI:** 10.3389/fonc.2016.00114

**Published:** 2016-05-06

**Authors:** Catherine Mercado, Marie-Adele Kress, Robyn A. Cyr, Leonard N. Chen, Thomas M. Yung, Elizabeth G. Bullock, Siyuan Lei, Brian T. Collins, Andrew N. Satinsky, K. William Harter, Simeng Suy, Anatoly Dritschilo, John H. Lynch, Sean P. Collins

**Affiliations:** ^1^Department of Radiation Medicine, Georgetown University Hospital, Washington, DC, USA; ^2^Department of Urology, Georgetown University Hospital, Washington, DC, USA

**Keywords:** prostate cancer, SBRT, CyberKnife, IMRT, EPIC, IGRT

## Abstract

**Purpose/objective(s):**

Stereotactic body radiation therapy (SBRT) is emerging as a minimally invasive alternative to brachytherapy to deliver highly conformal, dose-­escalated radiation therapy (RT) to the prostate. SBRT alone may not adequately cover the tumor extensions outside the prostate commonly seen in unfavorable prostate cancer. External beam radiation therapy (EBRT) with high dose rate brachytherapy boost is a proven effective therapy for unfavorable prostate cancer. This study reports on early prostate-specific antigen and prostate cancer-specific quality of life (QOL) outcomes in a cohort of unfavorable patients treated with intensity-modulated radiation therapy (IMRT) and SBRT boost.

**Materials/methods:**

Prostate cancer patients treated with SBRT (19.5 Gy in three fractions) followed by fiducial-guided IMRT (45–50.4 Gy) from March 2008 to September 2012 were included in this retrospective review of prospectively collected data. Biochemical failure was assessed using the Phoenix definition. Patients completed the expanded prostate cancer index composite (EPIC)-26 at baseline, 1 month after the completion of RT, every 3 months for the first year, then every 6 months for a minimum of 2 years.

**Results:**

One hundred eight patients (4 low-, 45 intermediate-, and 59 high-risk) with median age of 74 years completed treatment, with median follow-up of 4.4 years. Sixty-four percent of the patients received androgen deprivation therapy prior to the initiation of RT. The 3-year actuarial biochemical control rates were 100 and 89.8% for intermediate- and high-risk patients, respectively. At the initiation of RT, 9 and 5% of men felt their urinary and bowel function was a moderate to big problem, respectively. Mean EPIC urinary and bowel function and bother scores exhibited transient declines, with subsequent return to near baseline. At 2 years posttreatment, 13.7 and 5% of men felt their urinary and bowel function was a moderate to big problem, respectively.

**Conclusion:**

At 3-year follow-up, biochemical control was favorable. Acute urinary and bowel symptoms were comparable to conventionally fractionated IMRT and brachytherapy. Patients recovered to near their baseline urinary and bowel function by 2 years posttreatment. A combination of IMRT with SBRT boost is well tolerated with minimal impact on prostate cancer-specific QOL.

## Introduction

Prostate cancer has been shown to have a unique radiobiology resulting in a high sensitivity to fractionation (1). Analysis of clinical outcomes suggests that the α/β ratio for prostate cancer is approximately 1.5 Gy, rendering the tumor more biologically susceptible to large radiation fractions (2). Radiation dose escalation for prostate cancer has been shown to provide decreased biochemical failure rates and improved local control (3, 4).

High dose rate (HDR) brachytherapy, with its ability to deliver highly conformal large doses per fraction, has been used as a boost following external beam radiotherapy (EBRT) in the treatment of patients with intermediate- and high-risk prostate cancer with promising results (5). When compared to low dose rate (LDR) brachytherapy, its greater flexibility in dose delivery allows for improved coverage of extracapsular extension (ECE) and seminal vesicle invasion (SVI) (6, 7). Supplemental EBRT treats the prostate and seminal vesicles with a margin to encompass adjacent microscopic disease. Recent studies have illustrated 5-year biochemical control rates of 89–93 and 69–83% for intermediate- and high-risk prostate cancer patients, respectively (8–11). However, the administration of HDR brachytherapy is an invasive procedure requiring anesthesia and hospitalization, with potential risk of adverse events for elderly prostate cancer patients.

In an effort to provide an alternative method of dose escalation, stereotactic body radiation therapy (SBRT) is emerging as an alternative radiation therapy (RT) technique to deliver dose-escalated radiation to the prostate as a boost (12, 13). SBRT delivers highly conformal large radiation dose fractions *via* hundreds of non-isocentric beams to target volumes with precision (<1 mm) and steep dose gradients. In addition, SBRT incorporates a real-time tracking system that corrects the targeting of the therapeutic beam during treatment allowing for correction of intra-fraction motion. This allows for a reduction in the planning target volume (PTV) and, thus, minimizes radiation exposure to critical surrounding organs during treatment (14) resulting in a comparable toxicity profile to conventionally fractionated radiotherapy, despite higher doses per fraction (15, 16).

Currently, there are limited data to suggest that any particular treatment for early-stage prostate cancer is superior to another (17), therefore making the toxicity and side effect profile of an effective treatment critically important when choosing an intervention (18). Prostate cancer-specific quality of life (QOL) questionnaires are commonly utilized to assess these side effects, particularly the function and bother experienced by a patient posttreatment. The function domain measures the direct function and dysfunction of the urinary and bowel systems, while the bother domain measures a patient’s distress to the functional detriment of the urinary and bowel secondary to RT (18). Although it is necessary to evaluate functional decrements, bother may be of more importance when assessing patients’ QOL outcomes (18).

In this study, we present the 2-year QOL outcomes of 108 unfavorable prostate cancer patients treated with a combination of fiducial-directed intensity-modulated radiation therapy (IMRT) and SBRT boost with particular attention given to urinary/bowel function and bother.

## Materials and Methods

### Patient Selection

Patients eligible for inclusion in this study had histologically confirmed localized adenocarcinoma of the prostate treated with fiducial-guided IMRT and SBRT boost. Exclusion criteria included baseline prostate-specific antigen (PSA) >40 ng/ml, clinically involved lymph nodes, distant metastasis on imaging or bone scan, prior pelvic radiotherapy, and/or prior radical prostatectomy. Patient selection was accomplished by retrospective review of data that were prospectively collected in our institutional database. Institutional IRB approval was obtained for this study.

### Treatment Planning and Delivery

#### Stereotactic Body Radiation Therapy

Our SBRT methods have been described in detail previously (12). In brief, patients had four to six gold fiducials placed into the prostate prior to treatment planning. Patients underwent magnetic resonance imaging (MRI) 7 days after fiducial placement, followed by non-contrast simulation CT scan with 1.25 mm slice thickness. Both scans were performed with an empty bladder, and patients were advised to adhere to a low-gas, low-motility diet at least 5 days before imaging and treatment delivery. Patients took nothing by mouth the night prior to simulation, and an enema was administered 1–2 h before imaging and treatment. Fused MR and CT scan images were then used for treatment planning.

The clinical target volume (CTV) included the prostate, areas of radiographic ECE, and the proximal seminal vesicles to the point where the left and right seminal vesicles separate. The SBRT-PTV equaled the CTV expanded 3 mm posteriorly and 5 mm in all other dimensions. The rectum, bladder, and membranous urethra were contoured and evaluated. Treatment planning utilized Multiplan (Accuray Inc., Sunnyvale, CA, USA) inverse treatment planning for the course of SBRT. The target doses and dose constraints to critical surrounding anatomic structures have been described previously (12) and are summarized in Table 1.

**Table 1:** **Dose targets and constraints for supplemental SBRT treatment planning**.

19.50 Gy plan constraints	
PTV	*V* (19.5 Gy) ≥ 95%
Rectum	*V* (19.5 Gy) < l cc
	*V* (100%) < 5%
	*V* (90%) < 10%
	*V* (80%) < 20%
	*V* (75%) < 25%
	*V* (50%) < 50%
Bladder	*V* (19.5 Gy) < 5 cc
	*V* (100%) < 10%
	*V* (50%) < 40%
Membranous urethra	*V* (18 Gy) < 50%

Patients were treated with a SBRT prescription dose of 19.5 Gy to the PTV, which was delivered in three fractions of 6.5 Gy over 3–5 days using the CyberKnife Radiosurgical System (Accuray). The volume of the PTV receiving 19.5 Gy was at least 95%. The prescription isodose line was limited to >75%; the maximum prostatic urethra dose was limited to 133% of the prescription dose. Target position was verified multiple times during each treatment using paired, orthogonal X-ray images with a minimum of three properly placed fiducials (19).

#### Intensity-Modulated Radiation Therapy

Patients initiated IMRT the week following SBRT. The CTV for IMRT included the prostate, areas of radiographic ECE, and the proximal seminal vesicles. The IMRT-PTV included a margin of 1.0 cm around the CTV, except at the rectal interface, where a margin of 0.5 cm was added. The pelvic nodes were not treated. Daily doses of 1.8 Gy were delivered to PTV 5 days a week to a total dose of 45–50.4 Gy in 25–28 fractions. Eclipse planning system was utilized to design an inverse-planned course of IMRT. Daily image guidance was performed by matching gold fiducials. The minimum target dose constraint to the PTV was 98% and the maximum target dose constraint was 105% of the dose. In the delivery of IMRT, 100% of the PTV was to receive at least 95% of the prescription dose, and 5% of the volume was to receive no more than 105% of the prescription dose. For the bladder and rectum, the maximum dose constraint limit was 50 Gy, the full-volume dose constraint limit was 30 Gy and no part of either volume received more than 55.5 Gy. Dose to the femoral heads was limited to 45 Gy. The overall prescription dose to the PTV corresponded to a tumor equivalent dose in 2 Gy fractions (EQD2) of approximately 90 Gy assuming an alpha/beta ratio of 1.5.

### Pretreatment Assessment, Follow-up, and Statistical Analysis

Prostate-specific antigen levels were obtained and prostate cancer-specific QOL questionnaires were administered prior to the first SBRT treatment, 1 month after the completion of RT, every 3 months for the first year posttreatment, and every 6 months thereafter, for a minimum of 2 years. The expanded prostate cancer index composite (EPIC)-26 was used to evaluate urinary and bowel function and bother (17).

Differences in ongoing QOL scores were assessed and compared to baseline utilizing Wilcoxon signed rank test. The EPIC survey consisted of function and bother domains. The function domain measures the direct function and dysfunction of the urinary and bowel systems, while the bother domain measures a patient’s displeasure to the functional detriment secondary to RT. EPIC scores range from 0 to 100, with higher values representing a more favorable health-related QOL. The minimally important difference (MID) to assess for change in QOL scores was set as half a SD below baseline (20). Analysis of the QOL data included all time points that had at least an 80% patient response rate, which was up to 24 months for all QOL measures.

## Results

### Patients

From March 2008 to September 2012, 108 prostate cancer patients were treated per our institutional IMRT plus SBRT protocol. Table 2 provides a summary of patient characteristics. The median patient age was 74 years (range, 55–92 years). Similar numbers of White and Black patients were treated. The median pretreatment PSA was 9.1 ng/ml (range, 0.9–39.8 ng/ml). By D’Amico classification, 4 patients were low-, 45 intermediate-, and 59 high-risk. Seventy-eight percent of the patients were treated with an IMRT dose of 45 Gy in 25 fractions. Androgen deprivation therapy (ADT) was administered to 63.6% of patients for a median duration of 6 months (range, 3–36 months).

**Table 2:** **Patient characteristics and treatment**.

		No. of patients (*N* = 108)	(%)
Age (years)	Median 74 (55–92)		
Race	White	51	(47.2)
	Black	45	(41.7)
	Other	12	(11.1)
Pre-txt PSA (ng/ml)	Median 9.1 (0.86–39.8)		
T-stage	<T2a	50	(46.3)
	T2a–T2c	57	(52.8)
	≥T3	1	(0.9)
Gleason score	6 (3 + 3)	10	(9.3)
	7 (3 + 4; 4 + 3)	55	(50.9)
	8 (3 + 5; 4 + 4)	26	(24.1)
	9 (4 + 5; 5 + 4)	17	(15.7)
Risk groups (D’Amico’s)	Low	4	(3.7)
	Intermediate	45	(41.7)
	High	59	(54.6)
IMRT dose	45 Gy	84	(77.8)
	50.4 Gy	21	(19.4)
	Other	3	(2.8)
ADT	Yes	70	(63.6)
α_1A_ inhibitor		43	(39.8)

**Figure 1:**
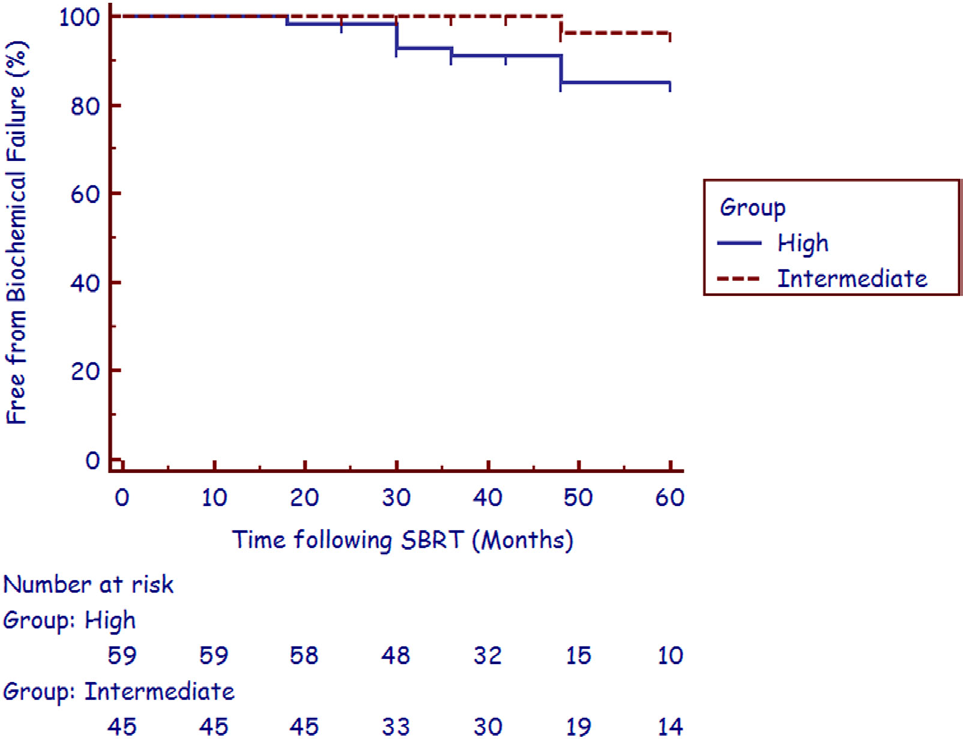
**Actuarial biochemical control for the intermediate-risk group and high-risk group**.

The median follow-up was 4.4 years (range, 2.1–6.8 years). There were nine biochemical failures per the Phoenix definition (21), occurring in one intermediate-risk patient and eight high-risk patients at a median of 36 months (range, 18–48 months). Three of the five post-failure prostate biopsies were positive for recurrent cancer. The corresponding 3-year actuarial biochemical control rates were 100 and 89.8% for intermediate- and high-risk patients, respectively (Figure 1). No patient died from prostate cancer during follow-up.

### Quality of Life

**Figure 2:**
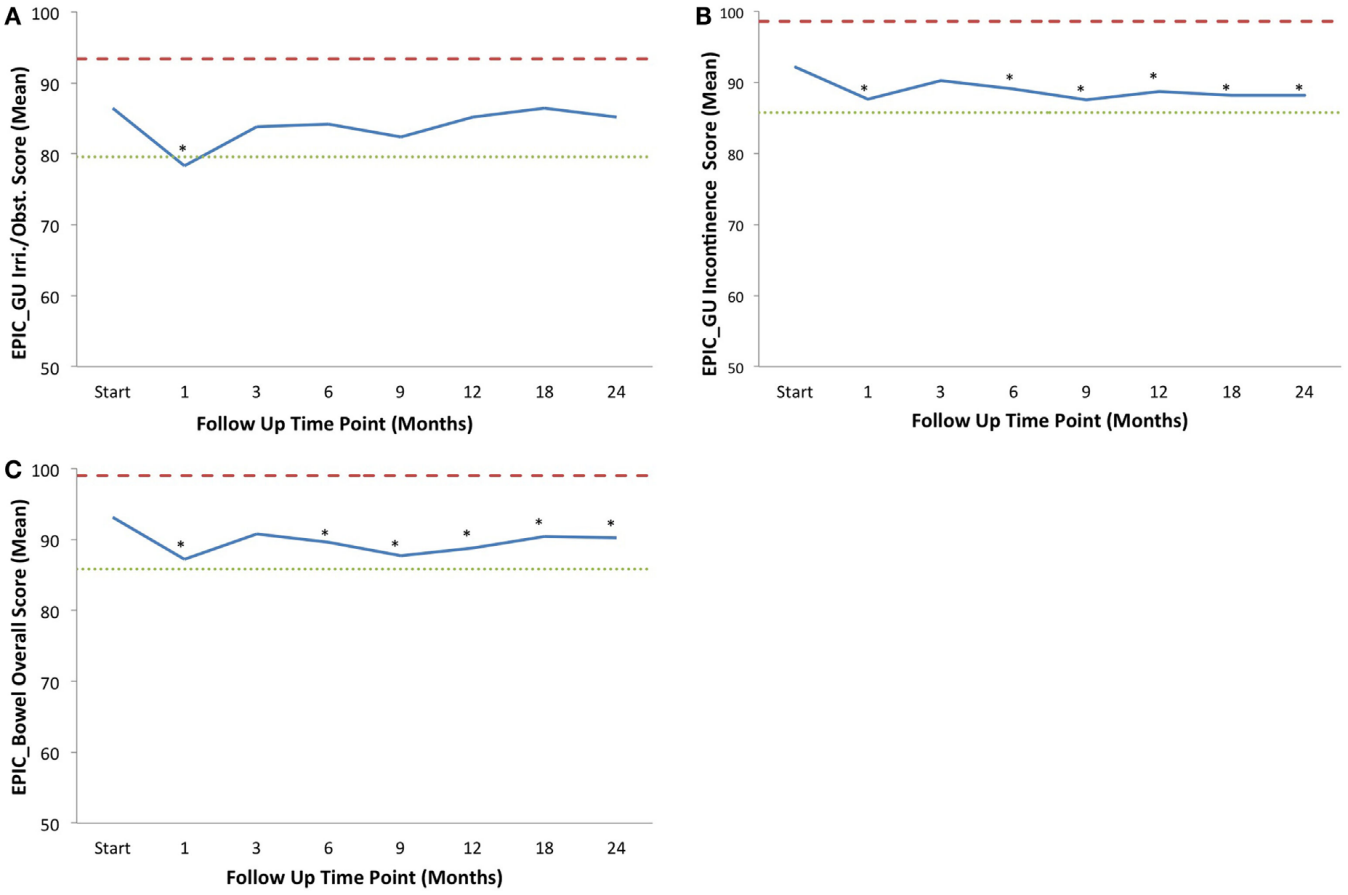
**Average EPIC domain scores at baseline and following IMRT plus SBRT boost for prostate cancer**. Shown are plots for: **(A)** EPIC urinary irritation/obstruction domain, **(B)** EPIC urinary incontinence domain, and **(C)** EPIC bowel domain. The thresholds for clinically significant changes in scores (½ SD above and below the baseline) are marked with dashed lines. IPSS scores range from 0 to 35 with higher values representing worsening urinary symptoms. EPIC scores range from 0 to 100 with higher values representing a more favorable health-related QOL. Thresholds for clinically significant changes in scores (½ SD above and below the baseline) are marked with dashed lines. EPIC scores range from 0 to 100 with higher values representing a more favorable health-related QOL.

Baseline EPIC summary scores are shown in Table 3 and mean changes in EPIC summary scores from baseline to 2-year follow-up are shown in Table 4. The EPIC scored urinary domain was stratified into two sub-domains: irritation/obstruction and incontinence. The mean urinary irritation/obstruction function score declined transiently at 1 month posttreatment (mean change from baseline, −8.2, *p* < 0.0001) and returned to baseline by 3 months post-RT (mean change from baseline, −2.6) (Table 4; Figure 2A). A second late decline in this function domain occurred at 9 months (mean change from baseline, −4.2, *p* = 0.17) with recovery by 12 months (*p* = 0.49). Only the decline at 1 month was statistically significant and met the threshold for clinically significant change (MID = 6.9). The EPIC irritation/obstruction function domain nearly returned to baseline by 24 months post-RT (mean change from baseline, −1.3, *p* = 0.55).

**Table 3:** **Pretreatment quality of life (QOL) scores**.

	Mean	SD	MID
EPIC-GU irritat./obstruct.	86.5	13.8	6.9
EPIC-GU incontinent	92.2	12.6	6.3
EPIC-bowel	93.1	14.6	7.3

**Table 4:** **Quality of life (QOL) domain scores over time**.

	1 month		3 months		12 months		24 months	
	Change	SD	Change	SD	Change	SD	Change	SD
EPIC-GU irritat./obstruct.	−8.2^a^	16.8	−2.6	13.9	−1.3	16.7	−1.3	16.4
EPIC-GU incontinent	−4.6^a^	15.4	−1.9	14.5	−3.4^a^	16.1	−4.0^a^	18.32
EPIC-bowel	−5.9^a^	17.4	−2.3	13.7	−4.3^a^	17.0	−2.9^a^	16.1

*EPIC, expanded prostate cancer index composite*.

*^a^Statistically significantly different from baseline (start)*.

Baseline irritative and obstructive urinary symptoms were common in this cohort of elderly men (Table 5). The most bothersome baseline symptoms were weak stream (moderate to big problem in 6%) and frequency (moderate to big problem in 12%) (Table 5). At 1 month post-RT, 9 and 21% of patients reported moderate to big problems with weak stream and frequency, respectively. A second late increase in irritative/obstructive symptoms occurred at 9 months with 22% of patients reporting a moderate to big problem with urinary frequency.

**Table 5:** **Percentage of patients reporting specific levels of distress or dysfunction**.

	Baseline *n* = 106	1 month *n* = 98	3 months *n* = 97	6 months *n* = 101	9 months *n* = 92	12 months *n* = 95	18 months *n* = 92	24 months *n* = 97
**Urinary function**								
Irritation or obstruction								
Dysuria^a^	1	4	2	5	8	5	2	4
Hematuria^a^	0	1	0	2	2	2	2	0
Weak stream^a^	6	9	8	4	5	6	2	3
Frequency^a^	12	21	11	14	22	15	13	11
Incontinence								
Leaking >l time per day	3	2	4	4	8	4	8	6
Frequent dribbling/no control	5	6	2	3	4	2	2	4
Any pad use	1	5	3	4	4	3	4	5
Leaking problem^a^	1	0	3	2	4	0	4	4
Overall urinary problem^a^	9	15	10	11	15	15	13	14
**Bowel function**								
Urgency^a^	3	8	8	11	10	8	7	6
Frequency^a^	3	7	6	9	3	6	5	6
Fecal incontinence^a^	1	4	2	2	3	5	3	4
Bloody stools^a^	1	4	0	0	0	3	2	2
Rectal pain^a^	6	4	0	2	4	2	1	2
Overall bowel problem^a^	5	8	5	9	12	10	3	5

*IMRT, intensity-modulated radiation therapy; SBRT, stereotactic body radiation therapy*.

*^a^Calculated based on item being “a moderate” or “big problem” on the EPIC QOL survey*.

The baseline EPIC urinary incontinence score and its mean changes from baseline to 2-year follow-up are shown in Tables 2 and 3. The mean score acutely declined at 1 month post-RT (mean change from baseline, −4.6, *p* = 0.02) and returned to near baseline by 3 months (mean change from baseline, −1.9, *p* = 0.13) (Table 4; Figure 2B). This change was statistically significant but of borderline clinical significance (MID = 6.4). EPIC urinary incontinence scores showed a second late protracted decline over the next 2 years. At 2 years post-RT, the mean summary score decreased from a baseline of 92.2 to 88.2 (mean change from baseline, −4.0) (Table 4; Figure 2B). This change was statistically significant (*p* = 0.02), but of borderline clinical significance (MID = 6.4).

Baseline urinary incontinence was uncommon in this patient cohort (Table 5). Prior to RT, 3% of patients reported leaking greater than once per day and 5% reported frequent dribbling or no control at all (Table 5). However, only 1% reported pad usage prior to RT. At 2 years post-RT, 6, 4, and 5% of patients reported incontinence based on the definitions of leaking greater than one time per day, frequent dribbling, and pad usage.

The baseline EPIC bowel summary score is shown in Table 3, and mean changes in EPIC bowel summary scores from baseline to 2-year follow-up are shown in Table 4. The mean bowel function score transiently declined from baseline to a nadir at 1 month post-RT (mean change from baseline, −5.9, *p* = 0.0001) with some recovery by 3 months (mean change from baseline, −2.3, *p* = 0.09) (Table 4; Figure 2C). A second decline occurred at 6 months (mean change from baseline, −3.5, *p* = 0.007) and remained low at 2 years posttreatment (mean change from baseline, −2.9, *p* = 0.014). From 6 to 24 months, the change in bowel function from baseline was statistically significant but of borderline clinical significance (MID = 7.3).

Baseline proctitis symptoms were uncommon in our patients (Table 5). All proctitis symptoms except rectal pain increased at 1 month post-RT. The most bothersome symptoms were bowel urgency and frequency. At 1 month post-RT, 8 and 7% of patients reported moderate to big problems with bowel urgency and frequency, respectively. Bother with proctitis symptoms remained elevated throughout the 12 months post-RT with moderate to big overall bowel problem reaching its peak at 12% at 9 months post-RT. By 18 months post-RT, the percentage of patients with moderate to big bowel problems had returned to baseline (Table 5).

## Discussion

Long-term patient survival is common after treatment for prostate cancer; therefore QOL is of paramount importance when selecting treatment options for patients. Outcome satisfaction, more closely related to bother than function, has been associated with long-term QOL (18). In an effort to further improve patient-reported outcomes following RT in the treatment of unfavorable prostate cancer, SBRT is emerging as viable alternative RT technique to HDR brachytherapy for boost delivery. SBRT delivers highly conformal large radiation dose fractions with dosimetric analysis suggesting that adequate dose is delivered to areas of potential ECE and the proximal seminal vesicles, most commonly seen in unfavorable patients (22). Although prospective studies are needed to confirm long-term tumor control, currently reported data illustrate that early biochemical control rates in unfavorable patients treated with a combination of IMRT and SBRT boost are comparable to results from studies of patients receiving HDR brachytherapy boost (8, 10, 11, 16). In studies of HDR boost treatment, 3-year biochemical control rates for ­intermediate- and high-risk patients range from 90 to 95% and 75 to 92%, respectively (8–11). This high biochemical ­relapse-free survival rate has been reproduced in our study with the utilization of SBRT boost, with a 3-year biochemical control rate for intermediate- and high-risk patients at 100 and 89.8%, respectively (Figure 1).

In the first 2 years posttreatment, impacts on urinary and bowel QOL were minimal. Our QOL results are comparable to recent studies comparing patients who underwent single-modality treatment with SBRT (23–28), radical prostatectomy, definitive EBRT, or brachytherapy (18). In our study, despite two-thirds of patients being treated with ADT, prostate-specific QOL scores remained high, demonstrating minimal impact of treatment-related side effects.

In this study, EPIC scores for bowel function and bother initially declined posttreatment but demonstrated recovery from symptoms by a 2-year follow-up (Table 3). A similar pattern of decline and recovery was witnessed for urinary function and bother (Table 3), with the exception of urinary incontinence (Figure 2B). Long-term urinary incontinence has been demonstrated to increase at, and after, 2 years posttreatment using many treatment modalities, including protons, conventionally fractionated EBRT (both 3-D conformal RT and IMRT), and prostatectomy (29–32). The long-term trend of increasing urinary incontinence with time demonstrated in these reports demonstrates the importance of continuing follow-up on this study’s patient cohort to determine the impact of possible long-term urinary functional detriment and bother. However, the minimally invasive combination of IMRT with SBRT boost is well tolerated among prostate cancer patients and still compares favorably with these alternative treatment modalities within the first 2 years posttreatment.

Until recently, there were limited data supporting the safety and efficacy of such SBRT monotherapy in the intermediate-risk population. Current publications by multiple single institutions used SBRT monotherapy regimens of 35–40 Gy, delivered to the prostate in four to five fractions, for intermediate-risk patients. Additionally, a pooled analysis from a multi-institutional consortium has shown a favorable 5-year biochemical disease-free survival of 84% in intermediate-risk patients (33, 34). Following the publication, these papers and the analysis of our own results, it is now our policy to treat intermediate-risk patients with SBRT alone.

For high-risk patients, there remains a concern that the tight clinical margins required to limit the normal tissue doses to the rectum with SBRT may not be adequate to treat the extent of ECE. While the planned posterior margin for SBRT is 3 mm, the actually treated posterior margin is commonly limited to 2 mm or less to maintain rectal tolerance (1 cc < 36 Gy) (7, 22). In these patients, the risk of ECE beyond 2 mm is approximately 40–70% (35). In many high-risk patients, the SVI extends beyond the proximal seminal vesicles (36) and distal seminal vesicle motion cannot be accounted for by intraprostatic fiducials (37). Thus, we await the mature results of ongoing trials treating high-risk patients with SBRT alone prior to recommending it for all but well selected high-risk patients (38).

Currently, it is our policy to not include pelvic node irradiation in the treatment of high-risk patients. Two randomized trials have been published questioning the benefit of treating pelvic lymph nodes in these patients (39, 40). A prior study with SBRT plus or minus conventional pelvic RT has shown significantly higher bowel toxicity associated with pelvic node treatment (41). Whether the use of pelvic IMRT can reduce bowel toxicity and improve the therapeutic ratio in select patients is an area of current investigation (13).

Although this study illustrates favorable QOL outcomes to support the utilization of SBRT boost for the treatment of prostate cancer, it does have several limitations as a result of the retrospective nature of the study. However, patients, in our study, were accrued consecutively, and all data were collected prospectively in a centralized database. The analysis of QOL outcomes were a combined majority of intermediate- and high-risk prostate cancer patients, stratifying data for each risk group was not performed.

## Conclusion

A combination of fiducial-guided IMRT and hypofractionated SBRT is well tolerated for the treatment of clinically localized prostate cancer, despite the delivery of dose-escalated radiation. Early PSA results suggest a biochemical control comparable to a combination of EBRT and HDR brachytherapy for intermediate- and high-risk prostate cancer patients. In the first 2 years posttreatment, impacts on urinary and bowel bother were minimal. Patients recovered to their baseline urinary/bowel QOL by 2 years following treatment.

## Author Contributions

CM, M-AK, and RC are lead authors who participated in manuscript drafting, table/figure creation, and manuscript revision. LC and TY aided in data collection and table/figure creation. SL and EB are the dosimetrists who contributed dosimetric data and figures. BC, AS, KH, SS, AD, and JL are senior authors who aided in drafting the manuscript and manuscript revision. SC is the corresponding author who initially developed the concept, drafted, and revised the manuscript. All authors read and approved the final manuscript.

## Conflict of Interest Statement

SC and BC serve as clinical consultants to Accuray Inc. The Department of Radiation Medicine at Georgetown University Hospital receives a grant from Accuray to support a research coordinator. The other authors declare that they have no competing interests.

## Abbreviations

ADT, androgen deprivation therapy; AUA, American Urological Association; CTC, common toxicity criteria; CTV, clinical target volume; EPIC, expanded prostate cancer index composite; GI, gastrointestinal; GTV, gross target volume; GU, genitourinary; IMRT, intensity-modulated radiation therapy; LUTS, lower urinary tract symptoms; PTV, planning target volume; QOL, quality of life; RT, radiation therapy; SBRT, stereotactic body radiation therapy.
